# Supplementary material for: Evaluation of a Cosmetic Formulation Containing Arginine Glutamate in Patients with Burn Scars: A Pilot Study
Source: Pharmaceutics. 2024 Sep 30;16(10):1283. doi: 10.3390/pharmaceutics16101283 (PMC11510376; doi:10.3390/pharmaceutics16101283)
Supplement: Supplementary file 1 [file pharmaceutics-16-01283-s001.zip › pharmaceutics-3109090-supplementary.pdf]

Evaluation of a Cosmetic Formulation Containing Arginine Glutamate in Patients with Burn Scars: A Pilot Study

HanBi Kim [1,†], InSuk Kwak [2,†], MiSun Kim [3], JiYoung Um [1], SoYeon Lee [1], BoYoung Chung [1], ChunWook Park [1], JongGu Won [3] and HyeOne Kim [1,*]

[1] Department of Dermatology, College of Medicine, Hallym University, Kangnam Sacred Heart Hospital, Seoul 07441, Republic of Korea; khmamy1029@naver.com (H.K.); ujy0402@hanmail.net (J.U.); minggijeook@gmail.com (S.L.); victoryby@naver.com (B.C.); dermap@hanmail.net (C.P.)

[2] Department of Anesthesiology and Pain Medicine, Burn Center, Hallym University Hangang Sacred Heart Hospital, Seoul 07247, Republic of Korea; kwak65joy@gmail.com

[3] LG Science Park R&D Center, LG Household & Healthcare (LG H&H), Seoul 07796, Republic of Korea; misunkim0407@gmail.com (M.K.); wjg8867@lghnh.com (J.W.)

* Correspondence: hyeonekim@gmail.com; Tel.: +82-2-829-5221; Fax: +82-2-832-3237

† These authors contributed equally to this work.

Figure legend

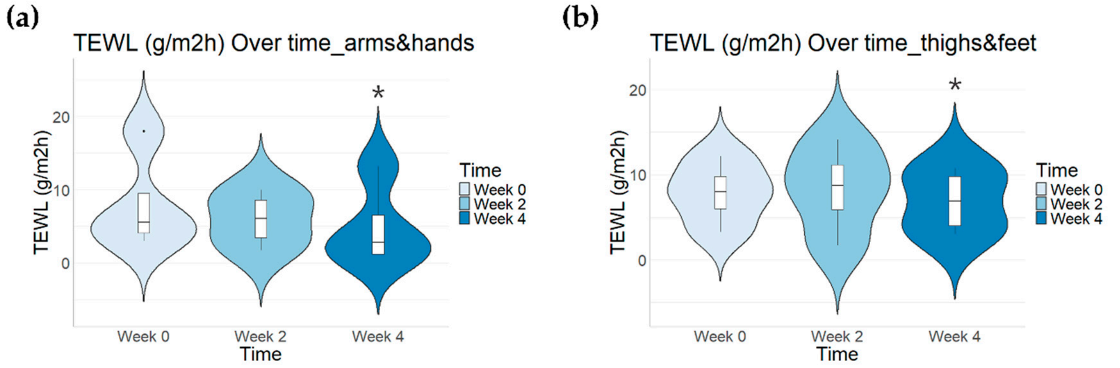

Supplementary Figure S1. Changes before and after treatment of TEWL (g/m2h) by skin area (n = 4). Data are represented as the mean value ± SD. (a) Arms&Hands, and (b) Thighs&Feet. (*$p < 0.05$ vs Week 0).

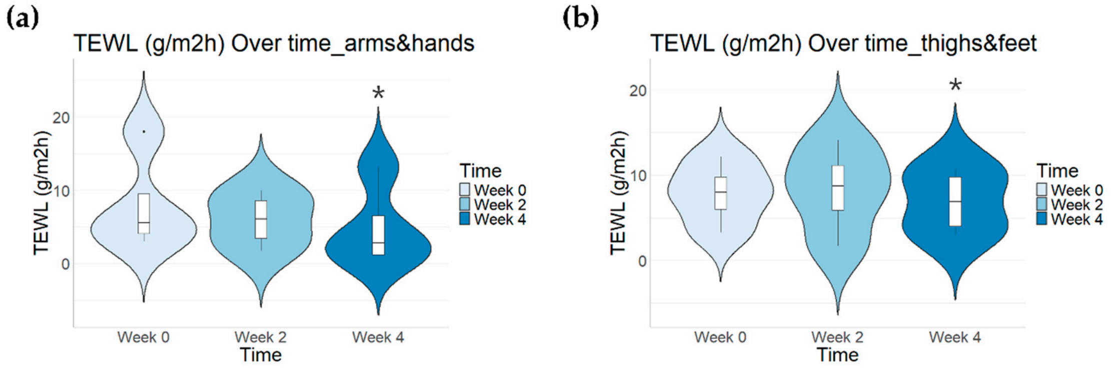

Supplementary Figure S2. Changes before and after treatment of SCH (A.U.) by skin area (n = 4). Data are represented as the mean value ± SD. (a) Arms&Hands, and (b) Thighs&Feet. (**p* < 0.05 vs Week 0).
